# The CHRNA3 rs578776 Variant is Associated with an Intrinsic Reward Sensitivity Deficit in Smokers

**DOI:** 10.3389/fpsyt.2013.00114

**Published:** 2013-09-23

**Authors:** Jason D. Robinson, Francesco Versace, Cho Y. Lam, Jennifer A. Minnix, Jeffrey M. Engelmann, Yong Cui, Maher Karam-Hage, Sanjay S. Shete, Gail E. Tomlinson, Tina T.-L. Chen, David W. Wetter, Charles E. Green, Paul M. Cinciripini

**Affiliations:** ^1^Department of Behavioral Science, The University of Texas MD Anderson Cancer Center, Houston, TX, USA; ^2^Department of Health Disparities Research, The University of Texas MD Anderson Cancer Center, Houston, TX, USA; ^3^Department of Biostatistics, The University of Texas MD Anderson Cancer Center, Houston, TX, USA; ^4^Division of Pediatric Hematology-Oncology, The University of Texas Health Science Center at San Antonio, San Antonio, TX, USA; ^5^Hamon Center for Therapeutic Oncology Research, The University of Texas Southwestern Medical Center at Dallas, Dallas, TX, USA; ^6^The University of Texas Medical School at Houston, Houston, TX, USA

**Keywords:** nAChR, DRD2, nicotine, reward sensitivity, ERP, LPP, smoking cessation, genetics

## Abstract

A compromised brain reward system has been postulated as a key feature of drug dependence. We examined whether several polymorphisms of genes found to regulate nicotinic acetylcholine receptor (nAChR) and dopamine expression were related to an intrinsic reward sensitivity (IRS) deficit we previously identified among a subgroup of smokers using event-related potentials (ERPs). We examined genetic polymorphisms within the *CHRNA5-A3-B4* gene cluster (*CHRNA3* rs578776, *CHRNA5* rs16969968, *LOC123688* rs8034191, and *CHRNA3* rs1051730), the *ANKK1* gene (rs1800497), and the D_2_ dopamine receptor gene (*DRD2* rs1079597, *DRD2* rs1799732) from 104 smokers of European ancestry in a smoking cessation trial. Prior to treatment, we recorded ERPs evoked by emotional (both pleasant and unpleasant), neutral, and cigarette-related pictures. Smokers were assigned to two groups (IRS+/IRS−) based on the amplitude of the late positive potential (LPP) component to the pictures, a neural marker of motivational salience. Smokers (*n* = 42) with blunted brain responses to intrinsically rewarding (pleasant) pictures and enhanced responses to cigarette pictures were assigned to the IRS− group, while smokers (*n* = 62) with the opposite pattern of LPP responding were assigned to the IRS+ group. Carriers of the protective minor T allele (T/T, C/T) of the *CHRNA3* rs578776 were less likely to be members of the IRS− group than those homozygous for the at-risk C allele (C/C). The *CHRNA3* rs578776 polymorphism did not differ on questionnaires of nicotine dependence, depressed mood, or trait affective disposition and did not predict abstinence at 6 months after the quit date. These results suggest that polymorphisms of genes influencing nAChR expression are related to an endophenotype of reward sensitivity in smokers.

## Introduction

A compromised brain reward system has been postulated as a key feature of drug dependence. Volkow and Colleagues (1–3) proposed that, due to the supraphysiological dopamine (DA) release in the midbrain resulting from drug use, addiction leads to overvaluing drug-related stimuli and to undervaluing intrinsically rewarding stimuli (e.g., food, sex), a condition we refer to as reduced intrinsic reward sensitivity (IRS−). The evidence for this differential salience is mixed. While many studies have found evidence for the enhanced motivational salience of drug-related stimuli among the drug dependent compared to controls (4–7), not all do (8, 9). Few studies have examined whether there is a concomitant reduction in the salience of intrinsically rewarding stimuli among the drug dependent. Of those that have, some have found that the drug dependent show enhanced salience to drug stimuli and reduced salience to intrinsically rewarding stimuli compared to controls (10–12), though other findings in the animal (13, 14) and human (15–17) literatures are equivocal.

Recently, using event-related potentials (ERPs), we found that smokers who demonstrated IRS− were more likely to relapse following a smoking cessation intervention than smokers with “normal” intrinsic reward sensitivity [IRS+; (18)]. We identified this endophenotype by analyzing smokers’ ERP differences to motivationally relevant visual stimuli. Baseline brain responses to unpleasant, neutral, pleasant, and cigarette-related (CIG) pictures were measured using the late positive potential (LPP). The LPP is an ERP component that peaks between 400 and 700 ms after stimulus onset and reliably indexes the motivational salience of passively viewed affective pictures (19–23). Consistent with the idea that after repeated drug use drug-related cues acquire motivational significance (2, 3, 24), smokers produce LPP responses to CIG pictures that are larger than those to neutral pictures (16, 25–27). However, by grouping smokers using individual patterns of brain reactivity, we discovered that while all smokers show larger LPP responses to cigarette than to neutral stimuli, a sizable number of them (45% of our sample) also show blunted brain responses to pleasant stimuli. Importantly, these individual differences in responsivity to pleasant stimuli predict subsequent smoking abstinence, such that smokers with blunted brain responses to intrinsically pleasant stimuli (IRS−) had significantly lower rates of long-term smoking abstinence than smokers with normal responses to pleasant stimuli (IRS+).

Our findings suggest that a large portion of smokers have blunted brain responses to intrinsically rewarding stimuli, but it is unclear why certain smokers have this presumed deficit while others do not. Genetic factors, particularly those that influence and regulate DA-mediated reward signaling, are likely targets. While nicotine dependence involves numerous neuroadaptations, the binding of nicotine to nicotinic acetylcholine receptors (nAChRs) on dopaminergic neurons in the ventral tegmental area (VTA) is thought to be key to nicotine’s effects on motivation (28). Nicotine binding on VTA dopaminergic neurons leads to increased DA burst firing, resulting in DA release in the outer shell of the nucleus accumbens (NAcc), a part of the mesolimbic reward pathway (29).

One possible source for this IRS− endophenotype is reduced striatal D_2_ DA receptor (D2R) density. A model that links striatal D2R deficits, D_2_ dopamine receptor (DRD2) (and ANKK1) genes, and substance abuse is the “reward deficiency syndrome” (RDS) model by Blum and Colleagues (30, 31). In the RDS model, individuals with deficits in *DRD2* receptor genes experience less reward and enjoyment from day-to-day activities (i.e., anhedonia) and are prone to substance dependence to increase DA levels and hedonic tone. The RDS model predicts that those with D2R deficits should experience greater enjoyment of drugs that stimulate DA release, which has been largely supported. For example, among drug-naive individuals, those with the lower striatal D2R density were more likely to report enjoying the novel effects of the psychostimulant methylphenidate (32, 33) compared with those with higher levels. However, research involving participants with chronic mental illness suggest that D2R deficits do not result in anhedonia, as suggested by the RDS model, but instead result in affective flattening (34, 35). Affective flattening is thought to reflect a reduction in the incentive salience of intrinsically pleasant stimuli and is consistent with the Incentive Salience model’s concept of “wanting” (24). This increase in drug reinforcement and decrease in responding to intrinsically rewarding cues appears to map onto the IRS− endophenotype and led us to examine whether genes that inform D2R expression could explain the differences we found.

The *DRD2* gene and the adjacent *ANKK1* gene have been linked to D2R deficits, nicotine dependence, and reward sensitivity. The *DRD2* gene is located on chromosome 11q22–q23 and three of the more frequently studied polymorphisms in the addictive disorders have been the *ANKK1* rs1800497, *DRD2* rs1799732 (−141C *Ins/Del*), and *DRD2* rs1079597 (*Taq*I-B). The *ANKK1* rs1800497, formerly known as the *DRD2 Taq*I-A, is a protein kinase gene located 9.5-kb downstream from the *DRD2* locus on chromosome 11 (36). Those carrying the T (A1) allele have fewer striatal D2R receptors compared to those without the T allele (37). The deletion variant of the *DRD2* rs1799732, located in the 5′ promoter region, has been associated with lower promoter activity (38). The biological significance of the *DRD2* rs1079597, located in intron 1, 913 bp from the exon 2 start codon, is unknown (39). The *ANKK1* rs1800497 has been linked to nicotine dependence in some (40, 41), but not all (42), studies. The *ANKK1* rs1800497, *DRD2* rs1079597, and *DRD2* rs1799732, have been found to predict the severity of smoking withdrawal (43, 44) and to moderate the effects of bupropion (45, 46) and nicotine replacement therapy [NRT; (46, 47)] on smoking cessation, though recent work has either failed to find a relationship between the *ANKK1* rs1800497 and smoking cessation outcome (42) or found a relationship with cessation only in subgroups such as women (48) or the depressed (49). In terms of the brain’s reward system, the *ANKK1* rs1800497 has been found to be related to smoking enjoyment (50) and to smoking with the intent of reducing negative affect (51), suggesting that it directly affects nicotine reinforcement.

Striatal DA functioning is impacted by nicotine (52) through its high affinity for α4β2-containing nAChRs in the VTA (53). Activation of the α3 (54), α4 (55), α6 (56), α7 (57), and β2 (58) nAChR subunits has been found to enhance nicotine reinforcement. In terms of intrinsic reward sensitivity (IRS), nicotine exposure has been found to lower the threshold for intrinsic reward for at least 30 days in rodents (59). In humans, indirect evidence comes from a study that found that schizophrenic smokers with greater baseline symptoms of affective flattening were more likely to benefit from treatment by varenicline, an α4β2 nAChR partial agonist, benefits that included increased abstinence and larger increases in reward sensitivity (60). The nAChR genes that have been most associated with smoking in many population studies are the *CHRNA5-A3-B4* gene cluster located on chromosome 15q25.1. The *CHRNA5* rs16969968 (a non-synonymous coding SNP in exon 5), the *CHRNA3* rs578776 (3′ UTR), the *CHRNA3* rs1051730 (a synonymous SNP in exon 5), and the rs8034191 (located in intron 2 of hypothetical gene *LOC123688*) are located in a region of strong linkage disequilibrium (LD) and have been found to be related to smoking and smoking heaviness (61–63). The *CHRNA5* rs16969968, the *CHRNA3* rs578776, *CHRNA3* rs1051730, and the *LOC123688* rs8034191 have all been found to be related to smoking rate and other measures of nicotine dependence (61–63). While none of the *CHRNA5-A3-B4* genetic subunits have been shown to directly influence functioning of the VTA or other areas associated with the brain’s reward system, recent evidence from transgenic mice found that overexpressing the human CHRNA5/A3/B4 gene cluster led to increased nicotine self-administration (64), suggesting a direct link between this cluster and nicotine reinforcement.

In the present study, we investigated whether *DRD2* and nAChR genetic variants known to influence striatal DA functioning and smoking behavior accounted for the reward sensitivity endophenotype we recently identified in smokers (18). We examined the relationship between this IRS endophenotype, comprised of ERP measures of motivational salience, and genetic polymorphisms within the *CHRNA5-A3-B4* gene cluster (*CHRNA3* rs578776, *CHRNA5* rs16969968, *LOC123688* rs8034191, and *CHRNA3* rs1051730), the *ANKK1* gene (rs1800497), and the D_2_ receptor gene (*DRD2* rs1799732, *DRD2* rs1079597), from 104 smokers of European ancestry in a smoking cessation trial [ClinicalTrials.gov Identifier: NCT00507728; (65)]. We hypothesized that smokers carrying at-risk nAChR, *ANKK1*, or D_2_ receptor alleles (i.e., alleles previously linked to smoking behavior and nicotine dependence) would be more likely to be in the IRS− group, as assessed by ERP to motivational stimuli, compared to those without these alleles. We also assessed the incremental impact of possessing multiple at-risk alleles on our ability to predict IRS group membership, using receiver–operator curve (ROC) analysis.

## Materials and Methods

### Participants

We recruited smokers from the community who were seeking to quit smoking. Inclusion criteria included being aged 18–65 years, smoking five or more cigarettes per day, producing a baseline expired carbon monoxide (CO) level ≥6 ppm, having fluency in English, and having a working telephone. Conditions that excluded participants included taking psychotropic medication, having a current psychiatric disorder (including substance abuse or dependence, other than nicotine dependence), being involved in any smoking cessation activities (e.g., NRT, counseling) except those provided by the clinical trial, having contraindications for either bupropion or varenicline, or having any uncontrolled medical illness. Psychiatric disorders were assessed using the Mini-International Neuropsychiatric Interview (66). Of the 294 enrolled in the clinical trial (65), 208 eligible participants completed a baseline laboratory ERP session, but only 180 yielded usable ERP data and were included in our previous report describing the reinforcement sensitivity construct (18). A total of 24 were excluded because of poor recording quality and 4 because of technical errors. Of those 180 from our previous study, 104 were of self-reported European ancestry and subject to the analyses described herein (data from the 49 participants of African ancestry differed significantly from those of European ancestry on several of the genotype frequencies and were excluded from further analyses). Participants provided written informed consent, and the protocol and informed consent document were approved by The University of Texas MD Anderson Cancer Center Institutional Review Board.

### Procedures

Participants were screened for eligibility a week before the baseline laboratory session. Participants were instructed to smoke *ad libitum* before the baseline laboratory session. At the baseline laboratory session, participants provided an expired carbon monoxide (CO) sample, provided a buccal sample, and completed questionnaires including the Fagerström Test for Nicotine Dependence [FTND; (67)], the Wisconsin Inventory of Smoking Dependence Motives [WISDM; (68)], the Center for Epidemiologic Studies Depression Scale [CES-D; (69)], the Depression Proneness Inventory [DPI; (70)], the Behavioral Inhibition System/Behavioral Activation System (BIS/BAS) scales (71) and the Fawcett–Clark Pleasure Scale [FCPS; (72)]. Next, the participants had the electroencephalogram (EEG) electrodes applied and were instructed to keep their eyes on the screen during the picture-viewing task, to move as little as possible, and to ignore the intermittent auditory startle probe noises delivered through the earphones during the picture viewing.

#### Picture-viewing task

During the 30-min picture-viewing task, participants viewed 1 of 3 picture sets composed of 4 picture categories, including pleasant (PLE), unpleasant (UNP), CIG, and neutral (NEU), with 24 pictures in each category (96 total pictures per set). The three picture sets were comprised of pictures from the International Affective Picture System (IAPS) (73), the International Smoking Image Series (ISIS) (74), and the Normative Appetitive Picture System (NAPS) (75). We supplemented these sets using pictures generated by our laboratory, some of which were previously published (76). Each set of 96 pictures included 24 pictures in 4 categories (PLE, UNP, NEU, CIG). Each picture category included 16 pictures depicting human beings and 8 without a person. In terms of semantic content, the PLE category was comprised of erotic couples (high arousal), romantic couples (low arousal), and pleasant objects (e.g., food, landscapes; low arousal), UNP category of mutilations (high arousal), sad scenes (e.g., grief, disease; low arousal), and unpleasant objects (e.g., pollution, accidents; low arousal), NEU category of neutral people and objects (e.g., household objects), and the CIG category of people smoking and CIG objects (e.g., lit cigarettes in ashtrays). The arousal ratings of the PLE and UNP pictures, which were primarily taken from the IAPS set (see Table 1), are from published IAPS normative ratings (73). Pictures were presented for 4 s, separated by a random inter-trial interval of 3–5 s, in pseudo-random sequences with no more than two pictures of the same category presented consecutively. Each picture was presented twice during the session to increase the ERP signal-to-noise ratio. Six (25%) the 24 pictures in each category included a 50-ms 100 dB (A) white noise auditory startle probe that occurred between 2.5 and 3.5 s after picture onset. These probes occur substantially after the ERP component of interest here (400–700 ms) and as such did not impact the ERPs to the pictures and hence are not discussed in this report. Stimuli were presented with a Pentium 4 computer using E-prime software (v1.4; Psychology Software Tools, Inc., Pittsburgh, PA, USA) on a plasma screen placed approximately 1.5 m from the participant’s eyes. The entire picture-viewing session lasted approximately 30 min.

**Table 1:** **List of pictures used in the picture-viewing task, by picture category and source**.

Source	PLE	UNP	NEU	CIG
IAPS	2208, 4599, 4610, 4611, 4623, 4624, 4625, 4626, 4640, 4641, 4643, 4645, 4647, 4649, 4650, 4653, 4658, 4659, 4660, 4666, 4669, 4676, 4677, 4680, 4687, 4689, 4690, 4691, 4693, 4694, 4695, 4696, 4698, 4700, 4800, 4810, 5250, 5626, 5628, 5629, 5631, 5660, 5661, 5700, 5711, 5764, 5780, 5781, 7270, 7330, 7340, 7350, 7410, 7430, 7450, 7460, 7470, 7480, 7482, 7485	2095, 2141, 2205, 2276, 2399, 2455, 2490, 2491, 2520, 2590, 2700, 2703, 2800, 2810, 2900, 3000, 3030, 3051, 3053, 3060, 3068, 3069, 3080, 3100, 3101, 3102, 3110, 3120, 3130, 3140, 3168, 3170, 3225, 3261, 3280, 3300, 3350, 6000, 6020, 6190, 6200, 6210, 6230, 6260, 6410, 9010, 9090, 9110, 9190, 9253, 9265, 9290, 9300, 9301, 9320, 9373, 9390, 9410, 9420, 9421, 9429, 9433, 9520, 9530, 9560, 9600, 9620, 9621, 9901, 9911, 9912, 9926	2102, 2104, 2190, 2191, 2200, 2210, 2214, 2215, 2220, 2221, 2230, 2235, 2305, 2312, 2358, 2372, 2383, 2393, 2396, 2397, 2435, 2441, 2485, 2493, 2495, 2499, 2500, 2510, 2512, 2513, 2515, 2570, 2575, 2579, 2593, 2594, 2595, 2597, 2598, 2600, 2630, 2830, 2850, 7000, 7002, 7004, 7006, 7009, 7010, 7020, 7030, 7034, 7038, 7040, 7041, 7052, 7053, 7054, 7055, 7056, 7059, 7080, 7090, 7110, 7130, 7140, 7550, 9070	
ISIS				001, 003, 004, 007, 008, 010, 016, 017, 019, 020, 022, 029, 030, 031, 033, 034, 038, 047, 055, 058, 062, 063, 068, 070, 073, 081, 088, 089, 091, 092, 093, 097, 099, 100, 101, 103, 104, 106, 107, 108, 111, 115, 116, 118, 120
NAPS				101, 106
Internal	(12 Pictures)	(0 Pictures)	(4 Pictures)	(25 Pictures)

IAPS, International Affective Picture System (73); ISIS, International Smoking Image Series (74); NAPS, Normative Appetitive Picture System (75). The internal pictures were generated by our laboratory, some of which were previously published (76), and are available upon request.

#### ERP data collection and scoring

Details of the EEG collection, offline scoring, and cluster analysis are reported in Versace et al. (18) and summarized here. We collected EEG during the picture-viewing task using a 129-channel EEG net and AC-coupled high-input impedance (200 MΩ) amplifier (Geodesic EEG System 200, Electrical Geodesics Inc., Eugene, OR, USA) referenced to the Cz electrode site. EEG was sampled at 250 Hz and filtered online using 0.1-Hz high-pass and 100-Hz low-pass filters, and the scalp impedance of each sensor was brought below 50 kΩ, as suggested by the manufacturer.

Offline scoring included the following procedures: (1) application of a 30-Hz low-pass filter; (2) visual inspection of the data, with channels contaminated by artifacts for more than 50% of the recording interpolated using spherical splines; (3) application of a spatial filtering method (77) to correct for eye blinks; (4) re-referencing to the average reference (78); (5) segmentation of the EEG data using a 900-ms epoch with a 100-ms baseline preceding the picture; (6) removal of segments if more than 10% of the sensors were contaminated by artifacts, defined as EEG amplitude above 100 or below −100 mV, voltage difference between any two adjacent samples larger than 100 mV, voltage difference between two adjacent samples above 25 mV, or variation of <0.5 mV for more than 100 ms; (7) calculation of the mean LPP amplitude between 400 and 700 ms after picture onset for each category (UNP, NEU, PLE, CIG) for each participant by averaging over 10 sensors in the central and parietal regions with the largest LPP differences between neutral and motivationally relevant (UNP/PLE/CIG) pictures. We analyzed the LPP within the 400- to 700-ms time window because it is considered the time window within which the LPP differences among categories peak and it is routinely used in studies investigating ERP responses to emotional and neutral stimuli (21, 22, 79–81). Moreover, increasing the length of the time window would have increased the number of epochs discarded because of the presence of artifacts, which could have reduced the reliability of the results without significantly improving the power to detect differences among the stimuli.

#### ERP cluster analyses

Details of the LPP cluster analysis are reported in Versace et al. (18). The four category LPP means from each participant were entered into a cluster analysis (*k*-means method) to assign smokers to two groups (IRS group) based on their brain responses to the experimental visual stimuli. Cluster analysis is a group of multivariate techniques whose purpose is to assemble objects (i.e., participants) based on the response patterns of several characteristics (82). A two-cluster solution was selected *a priori* because we hypothesized that smokers would differ on the basis of their sensitivity to intrinsically pleasant stimuli, which resulted in 99 participants assigned to Cluster 1 and 81 participants to Cluster 2. To evaluate if the two-cluster solution was appropriate, we used Euclidean distances and the Hartigan and Wong (83) algorithm for solutions ranging from 2 to 15 clusters. The Cubic Clustering Criterion (84) and the Duda–Hart Index (85), two commonly used stopping rules for iterative clustering, both indicated that the two-cluster solution was optimal. We next conducted an ANOVA using cluster (Cluster 1 vs. 2) as a between-subjects factor and picture type (UNP, NEU, PLE, CIG) as a within-subjects factor and found a significant two-way interaction, *F*(3,534) = 43.49, *p* < 0.0001. *Post hoc* contrasts indicated that LPP to pleasant pictures were significantly smaller in Cluster 2 (*M* = 1.76 *SE* = 0.17) than in Cluster 1 (*M* = 2.95, SE = 0.16; *p* < 0.0001), but that CIG pictures were somewhat larger LPP in Cluster 2 (*M* = 2.47, SE = 0.16) than in Cluster 1 (*M* = 1.81, SE = 0.15; *p* < 0.10). The two clusters did not differ on LPPs to either neutral or unpleasant pictures. We refer to Cluster 1 as the “normal” IRS cluster (IRS+) and Cluster 2 as the reduced IRS cluster (IRS−).

#### Genotyping

Genomic DNA was extracted from buccal cells by using the QIAmp DNA kit (cat. # 51185; QIAGEN Sciences, Valencia, CA, USA). Single nucleotide polymorphism genotyping of the DA and nAChR SNPs was performed using the 5′ nuclease assay to discriminate between the two alleles representing the different genotypes. The assay reagents for SNP genotyping consisted of a mix of PCR primers and TaqMan MGB probes (FAM and VIC labeled) that were obtained from Applied Biosystems (Foster City, CA, USA). TaqMan SNP Genotyping Assays (ABI) were used for the polymorphisms *ANKK1* rs1800497 (C_7486676_10), *DRD2* rs1079597 (C_2278884_10), *CHRNA5* rs16969968 (C_26000428_20), *CHRNA3* rs578776 (C_721253_10), *CHRNA3* rs1051730 (C_9510307_20), and *LOC123688* rs8034191 (C_479126_10). Probes and primer for the *DRD2* rs1799732 polymorphism were designed in-house (Probe 1: FAMTACCCGTTCCAGGCCMGBNFQ; Probe 2: VICTACCCGTTCAGGCCGMGBNFQ; Primer 1: AAACAAGGGATGGCGGAAT; Primer 2: CCCCACCAAAGGAGCTGTA). Each assay enables scoring of both alleles in a single well within a 384-well plate. All assays are optimized to work with genomic DNA and TaqMan Universal Master Mix from Applied Biosystems. Forty cycles of PCR were performed. Plates were analyzed using an ABI Prism 7900HT Sequence Detection System from Applied Biosystems.

#### Statistical analysis

The tests of Hardy–Weinberg Equilibrium (HWE) and LD, and frequencies by allele and genotype, were completed using SAS Proc Allele (v9.2; SAS Institute, Carey, NC, USA). We used chi-square tests to examine dominant (homozygous wild type vs. heterozygotes and homozygous variants) and recessive (homozygous variant vs. heterozygotes and homozygous wild type) models of gene action on IRS group membership, with the interpretation of statistical significance adjusted for multiple comparisons using the Bonferroni correction. We used *t*-tests to evaluate differences in baseline measures by genotype and IRS group, with Satterthwaite’s approximation for degrees of freedom when variance was unequal.

## Results

### Allele and genotype frequencies

The tests of HWE and LD, and frequencies by allele and genotype for the participants of European ancestry are reported in Table 2. The frequencies of the *DRD2* rs1079597 (*Taq*I-B) and *DRD2* rs1799732 (−141C Ins/Del) did not meet HWE criteria but were included in further analyses for exploratory purposes. In terms of LD, the *CHRNA5* rs16969968, *LOC123688* rs8034191, *CHRNA3* rs578776, and *CHRNA3* rs1051730 SNPs were all found to be in strong LD with each other (*r* range: 0.53–0.97). The *DRD2* rs1079597 was in LD with the *ANKK1* rs1800497, *r* = 0.96. The *CHRNB2* rs2072661 and *DRD2* rs1799732 were not in LD with the other SNPs.

**Table 2:** **Tests of Hardy–Weinberg equilibrium and frequencies by allele and genotype for the participants of European ancestry**.

Gene/SNP	Frequency		Hardy–Weinberg equilibrium		
Allele/genotype	*n*	%	χ^2^	*df*	*p* Value
*ANKK1* rs1800497			0.00	1	1.00
T	48	25.0			
C	144	75.0			
T/T	6	6.3			
T/C	36	37.5			
C/C	54	56.3			
*DRD2* rs1079597			8.46	1	0.004
A	59	30.7			
G	133	69.3			
A/A	3	3.1			
A/G	53	55.2			
G/G	40	41.7			
*DRD2* rs1799732			5.46	1	0.05
Ins	171	90.0			
Del	19	10.0			
Ins/Ins	79	83.2			
Ins/Del	13	13.7			
Del/Del	3	3.2			
*CHRNA5* rs16969968			1.17	1	0.39
A	71	35.2			
G	131	64.8			
A/A	10	9.9			
A/G	51	50.5			
G/G	40	39.6			
*CHRNA3* rs1051730			0.57	1	0.52
A	71	35.9			
G	127	64.1			
A/A	11	11.1			
A/G	49	49.5			
G/G	39	39.4			
*CHRNA3* rs578776			1.23	1	0.26
T	45	22.5			
C	155	77.5			
T/T	7	7.0			
C/T	31	31.0			
C/C	62	62.0			
*CHRNB2* rs2072661			0.87	1	0.43
A	50	25.0			
G	150	75.0			
A/A	8	8.0			
A/G	34	34.0			
G/G	58	58.0			
*LOC123688* rs8034191			0.98	1	0.40
C	70	35			
T	130	65			
C/C	10	10			
C/T	50	50			
T/T	40	40			

### Baseline characteristics, by IRS group

For this sample of 104 smokers of European ancestry, we assigned IRS group membership based on the cluster solution from our full (*n* = 180) sample (18). We analyzed our baseline questionnaires by IRS group, and found that IRS− smokers produced significantly higher expired CO values than IRS+ smokers, Satterthwaite *t*(60.43) = 2.03, *p* < 0.05 (see Table 3). The IRS groups did not differ on any other measure.

**Table 3:** **Baseline demographics, smoking, and affective characteristics, by IRS group (*n* = 104)**.

	IRS group	
Characteristic	IRS+	IRS−
***n* (%)**		
Total *n*	62 (59.6)	42 (40.4)
Female	17 (27.4)	16 (38.1)
Positive depression history	7 (11.3)	5 (9.5)
**MEAN (SD)**		
Age	44.34 (11.2)	44.95 (10.6)
Years of smoking	24.60 (12.0)	26.67 (10.8)
Cigarettes/day	21.13 (8.4)	21.79 (8.1)
Expired CO (ppm)	23.85 (9.1)	29.21 (15.42)^a^
FTND score	4.71 (2.3)	5.19 (2.2)
WISDM (total score)	44.45 (10.2)	46.35 (9.3)
CES-D (total score)	7.52 (6.1)	7.57 (6.2)
Depression proneness inventory	2.61 (1.0)	2.70 (1.1)
Fawcett–Clark Pleasure Scale	3.68 (0.4)	3.74 (0.3)
BAS	26.97 (5.8)	28.50 (4.8)
BIS	14.89 (3.5)	15.17 (2.9)

^a^ Significant *t*-test comparison, by IRS group, at α < 0.05 (uncorrected). CO, carbon monoxide; FTND, Fagerström test for nicotine dependence; WISDM, Wisconsin inventory of smoking dependence motives; CES-D, the center for epidemiologic studies’ depression scale; BAS, behavioral approach system; BIS, behavioral inhibition system.

### LPP amplitude, by IRS group and picture category

**Figure 1:**
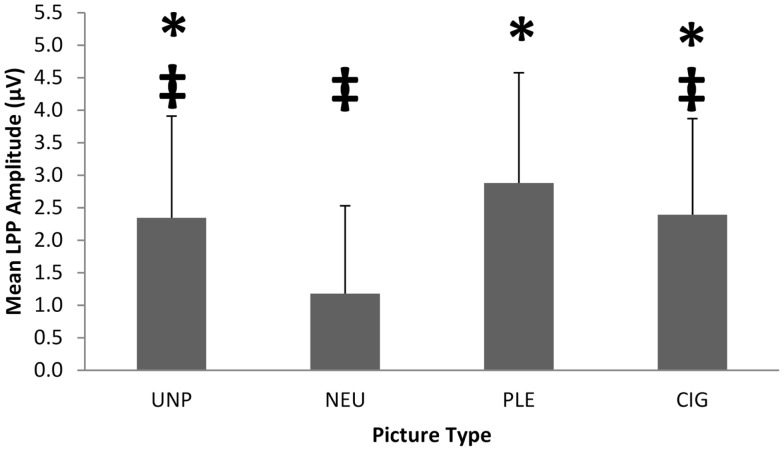
**The significant picture category (UNP, NEU, PLE, CIG) main effect on mean LPP amplitude for 104 smokers of European ancestry**. The error bars depict standard deviations. *Significantly different from NEU. ^‡^Significantly different from PLE.

**Figure 2:**
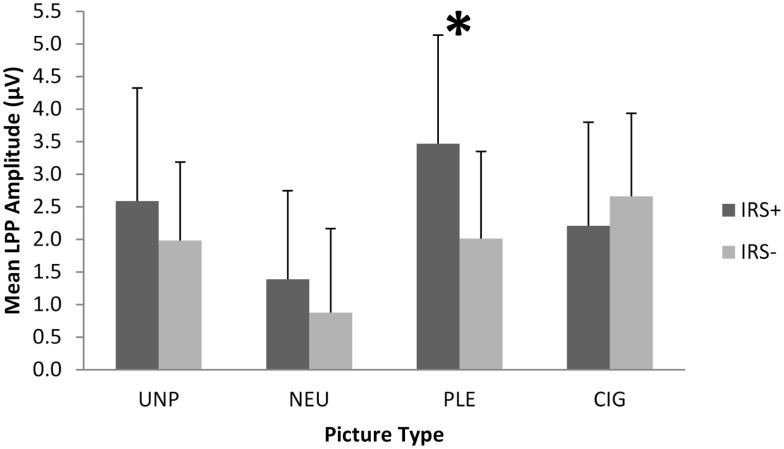
**The IRS group (IRS+ vs. IRS−) by picture category (UNP, NEU, PLE, CIG) interaction on mean LPP amplitude**. The error bars depict standard deviations. *Significant pairwise comparison,*p* < 0.0001.

To verify the differential responsivity by IRS group to cigarette and pleasant pictures in the present sample, we conducted an ANOVA on the 104 smokers of European ancestry using IRS group [IRS+ (*n* = 62) vs. IRS− (*n* = 42)] as a between-subjects factor, picture category (UNP, NEU, PLE, CIG) as a within-subjects factor, and mean LPP amplitude (between 400 and 700 ms after picture onset) as the dependent variable. We found a significant main effect of picture category, *F*(4,415) = 20.98, *p* < 0.0001 (see Figure 1), and two-way IRS group by picture category interaction, *F*(3,408) = 15.52, *p* < 0.0001 (see Figure 2). Comparable to the results from the full sample of 180 smokers (18), the *post hoc* contrasts for the 104 smokers of European ancestry indicated that LPPs to pleasant pictures were significantly smaller in IRS− smokers (*M* = 2.01 μV, SD = 1.34), than in IRS+ smokers (*M* = 3.47 μV, SD = 1.67; *p* < 0.0001). LPPs to CIG pictures were somewhat larger in IRS− (*M* = 2.66 μV, SD = 1.27) than in IRS+ smokers (*M* = 2.21 μV, SD = 1.59), but the difference was not statistically significant (*p* < 0.13). The two IRS groups did not differ on LPPs to either neutral or unpleasant pictures. Covarying expired CO did not alter the significant findings.

### Gene by IRS group

**Figure 3:**
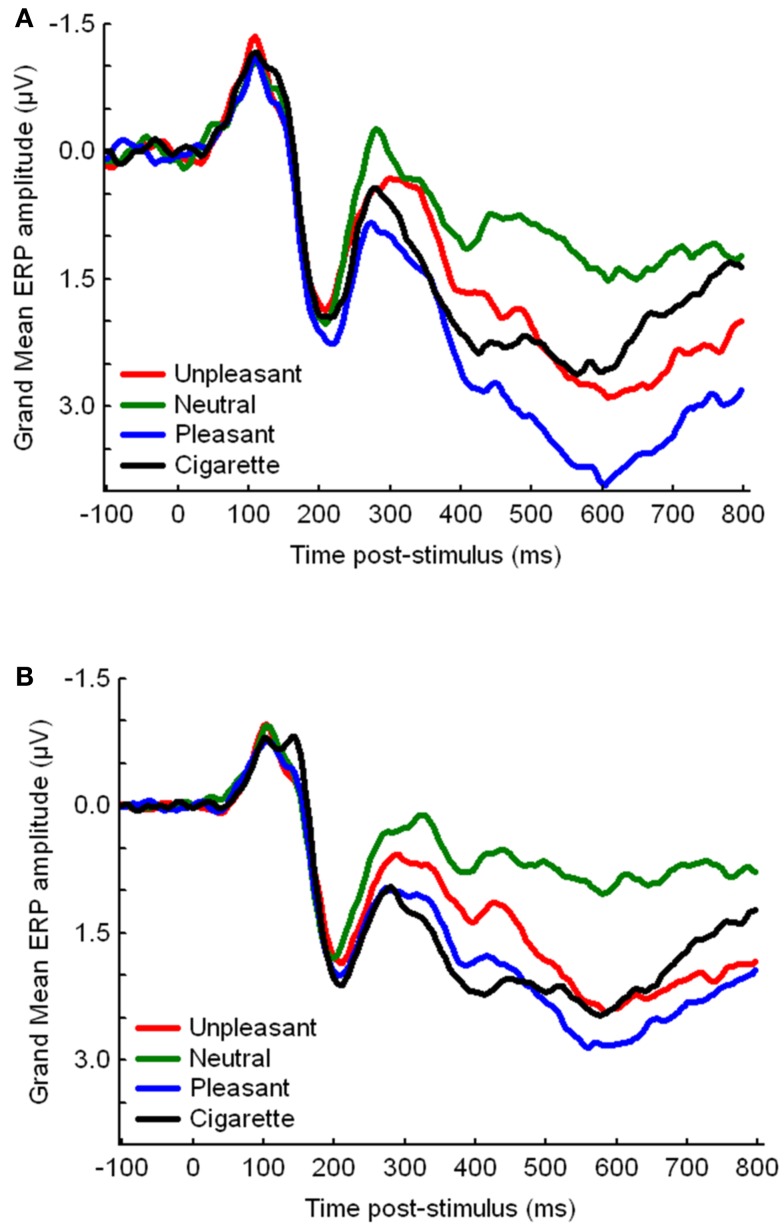
**Event-related potential waveforms, by picture category, for *CHRNA3* rs578776 (A) carriers of the protective minor T allele (T/T, C/T) and (B) those homozygous for the at-risk C allele (C/C)**.

The ORs and 95% CIs for IRS group membership by genotype (dominance models) are depicted in Table 4. After correcting for multiple comparisons, only the *CHRNA3* rs578776 was significant, with carriers of the protective minor T allele (T/T, C/T) less likely to be in the IRS− group than those homozygous for the at-risk C allele (C/C), OR = 0.17, 95% CI 0.07–0.46 (see Figure 3 for *CHRNA3* rs578776 ERP waveforms). Carriers of the at-risk T (A1) minor allele (T/T, T/C) of the *ANKK1* rs1800497 were more likely to be in the IRS− group than those homozygous for the C (A2) allele (C/C), OR = 2.39, 95% CI 1.04–5.52, but this difference was not significantly different after correcting for multiple comparisons. The *CHRNA5* rs16969968’s relationship to IRS group approached statistical significance prior to correcting for multiple comparisons, OR = 2.26, 95% CI 0.97–5.24, suggesting that carriers of the at-risk A minor allele (A/A, A/G) were more likely to be in the IRS− cluster than those homozygous for the G allele. The frequencies of the *LOC123688* rs8034191, *CHRNA3* rs1051730, DRD2 rs1079597, and *DRD2* rs1799732 SNPs did not significantly differ by IRS group.

**Table 4:** **Chi-square analyses with dominance models (homozygous wild type vs. heterozygotes and homozygous variants) predicting reduced intrinsic reward sensitivity (IRS− membership), all *df* = 1**.

Polymorphism	OR	95% CI	χ^2^	Uncorrected *p* value
*CHRNA3* rs578776	0.17	0.07–0.46	13.99	0.0002^a^
*ANKK1* rs1800497	2.39	1.04–5.52	4.28	0.04
*CHRNA5* rs16969968	2.26	0.97–5.24	3.66	0.06
*CHRNA3* rs1051730	1.97	0.84–4.60	2.48	0.12
*LOC123688* rs8034191	1.36	0.60–3.09	0.55	0.46
*CHRNB2* rs2072661	0.90	0.40–2.01	0.07	0.80
*DRD2* rs1079597	1.40	0.60–3.28	0.62	0.43
*DRD2* rs1799732	1.23	0.41–4.14	0.14	0.71

^a^ Statistically significant after correcting for multiple comparisons.

To determine whether the at-risk alleles have an additive impact on the prediction of IRS group membership, we examined the incremental impact of the other polymorphisms we evaluated on the *CHRNA3* rs578776’s ability to predict IRS group membership, using ROC analysis (86). As reported above, the presence of the protective T allele decreased the odds of being in the IRS−group by a factor of 0.17 (OR = 0.17, 95% CI 0.07–0.46). This corresponded to an area under the curve (AUC) of 0.68. Adding the *ANKK1* rs1800497 and *CHNRA3* 1051730 polymorphisms, as a block, incremented the fit of the model [Δχ^2^(2) = 6.32, *p* < 0.04] and increased the AUC to 0.76. These results show that the *CHRNA3* rs578776 predicted the IRS endophenotype, and that this prediction may be augmented by adding other SNPs associated with smoking behavior. Including the other polymorphisms did not significantly increase the AUC.

### Prediction of abstinence

In the original sample of 180, we found that IRS group predicted abstinence outcome (18), such that IRS− smokers quit smoking less often than did IRS+ smokers. In the current sample of 104 smokers of European ancestry, we verified this relationship using logistic models to compare the smoking abstinence rates of the two IRS groups at 10 weeks, 3 months, and 6 months after the quit date. In this reduced sample, IRS− smokers were also abstinent less often than IRS+ at 10 weeks (OR = 2.73, 95% CI 1.07–6.91), 3 months (OR = 3.06, 95% CI 1.19–7.90), and 6 months (OR = 4.03, 95% CI 1.37–11.88) after the quit date. There were no significant relationships between any of the SNPs (dominance models) and abstinence at 10-weeks (EOT), 3-months, or 6-months post-quit. There were also no significant interactions between IRS group and SNPs when predicting abstinence at any of the time points.

### Baseline measures, by genotype

To determine whether the participants differed on smoking or affective disposition by genotype, we analyzed our baseline questionnaires by the dominance models for the polymorphisms that differed by IRS group, the *ANKK1* rs1800497 and the *CHRNA3* rs578776, as well as the *CHRNA5* rs16969968. No significant differences (*p*s > 0.05) between genotype were found for any of the baseline questionnaires.

## Discussion

These results support the hypothesis that genes associated with nAChR activity are related to a novel neural marker of reward sensitivity in smokers. Carriers of the protective minor T allele of the CHRNA3 rs578776, a SNP in the CHRNA5-A3-B4 gene cluster on chromosome 15, were significantly less likely to be among those in the IRS− group. Though not statistically significant after correcting for multiple comparisons, carriers of the at-risk *ANKK1* rs1800497 T (A1) allele or the at-risk *CHRNA5* rs16969968 A allele were more likely to be among those in the IRS− group.

Our finding that the protective T allele of the *CHRNA3* rs578776 polymorphism is associated with IRS+ smokers is consistent with previous findings concerning the relationship between that allele and nicotine dependence. The protective minor T allele of the *CHRNA3* rs578776 has been found to be associated with reduced risk for nicotine dependence in European-Americans (87), but not African-Americans (88), and in smokers who began daily smoking before the age of 17 (89). The protective minor T allele of the *CHRNA3* rs578776 has also been found to be protective against heaviness of smoking (61, 90). In an fMRI study, the rs578776 at-risk C allele was associated with increased activation in a circuit comprising the dorsal anterior cingulate cortex and left anterior thalamus, a circuit that may be sensitive to nicotine exposure or to the alleviation of craving (91).

Our results suggest that the *CHRNA3* rs578776 T allele’s protective effect against smoking dependence may be due to its association with normal reward sensitivity to intrinsically pleasant activity in smokers. However, none of the baseline measures of nicotine dependence, depressed mood, or trait affective disposition differed by *CHRNA3* rs578776 genotype in the present study. This is unsurprising given that none of these questionnaires differed by IRS group in this and the full samples (18). This suggests that the IRS grouping represents a distinct endophenotype that is not easily captured by self-report and that offers clinical utility as a diagnostic marker of genetic and treatment risk. As we concluded in our previous manuscript (18), this endophenotype could be used to identify smokers who are at greater risk for relapse and who may need cessation interventions tailored to address their hyposensitivity to intrinsically reinforcing stimuli.

There were several null findings that were unexpected. First, some of the polymorphisms most commonly associated with smoking behavior, the *ANKK1* rs1800497, *CHNRA3* 1051730, and *CHRNA5* rs16969968, were not significantly related to IRS group. However, the at-risk alleles for these polymorphisms were associated with IRS− membership in the expected direction. They were likely not significant due to inadequate statistical power to detect their weaker effect size compare to that of the *CHRNA3* rs578776. Additionally, our ROC analysis found that the likelihood of IRS− group membership significantly increases with possession of the *ANKK1* rs1800497 and *CHNRA3* 1051730 at-risk alleles, suggesting that these polymorphisms are related to the endophenotype. Second, none of the polymorphisms predicted abstinence outcome, despite the significant associations between IRS group and abstinence at 10-weeks (EOT), 3-months, and 6-months post-quit. These findings are not necessarily inconsistent, because an endophenotype such as IRS group membership is likely determined by multiple genetic and environmental factors, as is shown by our ROC analysis.

Previous results largely support models of addiction that postulate that chronic drug use leads to overvaluing drug-related stimuli and undervaluing intrinsically reinforcing stimuli due to the large differential in DA enhancement between the two reinforcers (1–3). However, in a previous study (18), we found that this change in intrinsic reward valuation is not common to all chronic drug users. Our current results suggest that the individual’s genetic profile contributes to this variability. Our IRS endophenotype is consistent with Blums’ RDS model (30, 31) in that a subset of smokers appear to experience increased salience to drug-related stimuli and decreased salience to intrinsically rewarding stimuli. The individual differences reflected in the IRS endophenotype could explain the equivocal findings in the literature, with some (13, 15) but not all (14, 92, 93) studies finding that chronic substance users overvalue the drug compared to natural reinforcers. However, we were unable to conclusively link the IRS− deficit to the reduced D2R density predicted by the RDS model because of the statistically non-significant association between IRS group and the *ANKK1* rs1800497 polymorphism.

These findings are tempered by several limitations of this study. First, while the sample size was large for an ERP study, it was small for a genetic association study. This may explain why two other SNPs linked with smoking behavior, the *ANKK1* rs1800497 and the *CHRNA5* rs16969968, produced results suggesting a link between their at-risk variant and blunted brain responses to the intrinsically pleasant pictures that were statistically non-significant, after a correction for multiple comparisons. However, this limitation will likely confront any researcher who seeks to examine drug use endophenotypes that are more complex than smoker/non-smoker or nicotine dependence status. Second, we did not include a control group and only measured ERP during conditions of relative nicotine satiation, at baseline. We were thus unable to determine whether the reward system alterations we observed were specific to drug dependence or whether they would be exacerbated by protracted withdrawal in this study, as is predicted by recent addiction models [e.g., Ref. (2)]. Third, we were unable to separate out the pharmacological effects from the behavioral effects of smoking on LPP response, an issue that could be addressed by the use of blinded placebo cigarettes. Finally, it is possible that these genotypes we identified are markers for individuals with trait-level dysphoria or anhedonia, with smoking merely being a means by which these individuals “self-medicate” for deficiencies in their brain’s DA-mediated reward system (30). However, the participants in our sample did not differ by genotype on several questionnaire measures that are presumably sensitive to trait-level differences in affective predisposition. Indeed, the lack of association between the genotypes and our baseline measures of smoking dependence and trait affective disposition strengthens the clinical relevance of our IRS group measure and highlights the potential relevance of the LPP grouping as an endophenotype for predicting treatment response.

Future studies should examine whether the IRS− endophenotype is found among those with other drug abuse disorders and those from a healthy population to determine the extent to the IRS− endophenotype represents a risk factor specific to addiction. The relationship between this endophenotype and the development of nicotine dependence should be examined among adolescents at risk for nicotine dependence. This endophenotype should be replicated and extended by using imaging technology to more precisely identify related regions of the brain. The relationship between IRS group and other endophenotypes related to reward, such as impulsivity (94), should be investigated. Finally, the relationship between the IRS group endophenotype and other genetic polymorphisms related to the brain’s reward system and nicotine dependence should be examined, including dopamine D4 receptor (*DRD4*) (95) and DA transporter (*SLC6A3*) (96) genes.

## Conclusion

In conclusion, these results suggest that polymorphisms of genes influencing nAChR expression are related to a neural marker of reward sensitivity in smokers, a marker that may be a clinically useful endophenotype for identifying smokers at risk for relapse. In particular, the *CHRNA3* rs578776 was found to predict membership in clusters that varied by reward sensitivity to natural and cigarette stimuli and that predicted smoking cessation outcome. These results suggest that an endophenotype comprised of ERP measures of motivational salience can be a valuable tool for studying the interplay between drug dependence, genetics, and brain alterations thought to maintain dependence and as a diagnostic marker for identifying smokers who are at greater risk for relapse and who may need cessation interventions tailored to address their hyposensitivity to intrinsically reinforcing stimuli.

## Conflict of Interest Statement

This study was sponsored by the National Institute on Drug Abuse through grant R01 DA017073 to Paul M. Cinciripini. Other support included a NIDA sponsored K23 DA024697 to Jason Robinson, and a faculty fellowship from The University of Texas MD Anderson Cancer Center Duncan Family Institute for Cancer Prevention and Risk Assessment to Francesco Versace and the National Institutes of Health through MD Anderson’s Cancer Center Support Grant (P30 CA016672). Dr. Paul M. Cinciripini served on the scientific advisory board of Pfizer Pharmaceuticals, conducted educational talks sponsored by Pfizer on smoking cessation (2006–2008), and has received grant support from Pfizer. Dr. Karam-Hage has conducted educational talks sponsored by Pfizer Pharmaceuticals, and has participated as study physician and co-investigator in two studies funded by Pfizer Pharmaceuticals. In 2011, Dr. Versace received an independently reviewed competitive grant supported by Pfizer (Global Research Award for Nicotine Dependence). The other authors declare no conflict of interest.
